# Multiple Transverse Colonic Perforations Associated with Slow-Release Nonsteroidal Anti-Inflammatory Drugs and Corticosteroids: A Case Report

**DOI:** 10.1155/2011/824639

**Published:** 2011-07-12

**Authors:** Nobuki Shioya, Shigehiro Shibata, Masahiro Kojika, Shigeatsu Endo

**Affiliations:** Department of Critical Care and Emergency, Iwate Prefectural Advanced Critical Care and Emergency Center, Iwate Medical University, 19-1 Uchimaru, Morioka 020-8505, Japan

## Abstract

The patient was a 36-year-old woman with sarcoidosis and Sjogren's syndrome, and had been prescribed slow-release diclofenac sodium and prednisolone for the treatment of pain associated with uveitis and erythema nodosum. She was admitted to our emergency center with abdominal pain and distention. A chest X-ray showed free air under the diaphragm on both sides, and an emergency laparotomy was performed for suspected panperitonitis associated with intestinal perforation. Laparotomy revealed several perforations on the antimesenteric aspect of the transverse colon. The resected specimen showed 11 punched-out ulcerations, many of which were up to 10 mm in diameter. The microscopic findings were non-specific, with leukocytic infiltration around the perforations. She showed good postoperative recovery, as evaluated on day 42. The present case highlights the need for exercising caution while prescribing slow-release nonsteroidal anti-inflammatory drugs with corticosteroids to patients with autoimmune diseases, as such treatment may exacerbate intestinal epithelial abnormalities.

## 1. Introduction

Multiple intestinal perforations, although rare, may be fatal and may be caused by Crohn's disease, Behcet's disease, systemic lupus erythematosus (SLE), medications, infections, and neoplasms. Several autoimmune diseases are associated with changes in the intestinal permeability, and increased colonic permeability induces ulceration and perforation. Sarcoidosis and Crohn's disease share many clinical and immunological features and also have similar pathogenetic mechanisms [1, 2]; Sjogren's syndrome and sarcoidosis also have much in common in terms of the pathophysiology. However, there are no case reports of multiple perforations clearly associated with Sjogren's syndrome and sarcoidosis in the literature. Nonsteroidal anti-inflammatory drugs (NSAIDs) and corticosteroids are commonly prescribed worldwide for a broad spectrum of autoimmune manifestations. NSAIDs are known to damage the surface epithelial cells and increase colonic permeability, and concomitant use of NSAIDs and corticosteroids is known to cause additive damage to the gastrointestinal (GI) tract. Herein, we describe a rare case of multiple localized perforations of the transverse colon associated with coadministration of a slow-release NSAID and low-dose corticosteroid.

## 2. Case Presentation

A 36-year-old Japanese woman was referred to our critical care and emergency center with mild abdominal distention and epigastric pain of about 10 days duration. She gave a history of having been diagnosed as having bilateral uveitis and erythema nodosum in the lower extremities six months previously. The patient was diagnosed as having Sjogren's syndrome based on a positive Schirmer's test and reduced amount of saliva. During the 2 months prior to hospitalization, the patient suffered from loss of vision, ophthalmoplegia, and painful erythema nodosum nodules in the lower extremities. She had taken prednisolone at the dose of 20 mg/day. The serum albumin and calcium levels were reduced (2.4 mg/dL and 6.7 mg/dL). The serum angiotensin-converting enzyme (ACE) and lysozyme levels were slightly elevated (38.4 IU/l and 23.2 ug/mL, resp.). The tuberculin skin test was the reaction with induration of less than 5 mm in size. Bronchoalveolar lavage contained 61% lymphocytes, with a CD4/CD8 ratio of 4.9, and examination of transbronchial lung biopsy (right B8) specimens revealed the presence of noncaseating epithelioid cell granulomas. Microscopic examination of PAS- and acid-fast stained specimens revealed no acid-fast bacilli. Based on these findings, except for the low serum levels of calcium, sarcoidosis had been diagnosed about 4 months previously. She had suffered from pain of progressively increasing severity because of uveitis and erythema nodosum during the 10 days prior to admission. She had been prescribed 75 mg/day of slow-release diclofenac sodium and prednisolone in slowly tapering doses (current dose, 5 mg/day).

**Figure 1:**
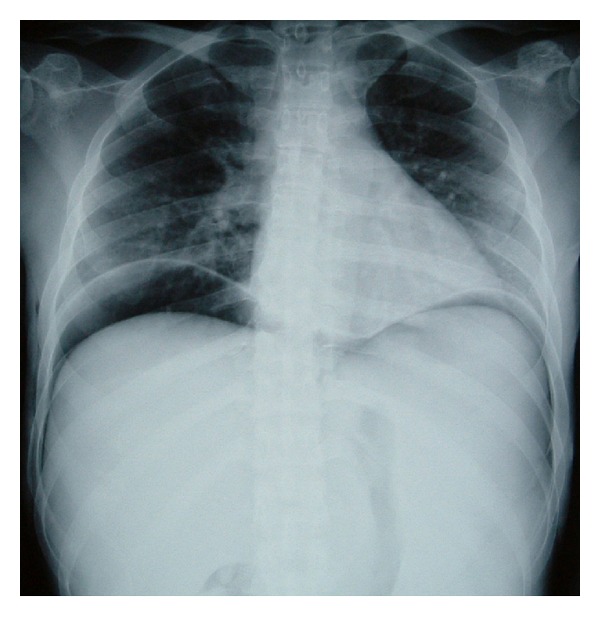
Chest radiograph at the initial diagnosis showed bilateral hypodiaphragmatic free air.

**Figure 2:**
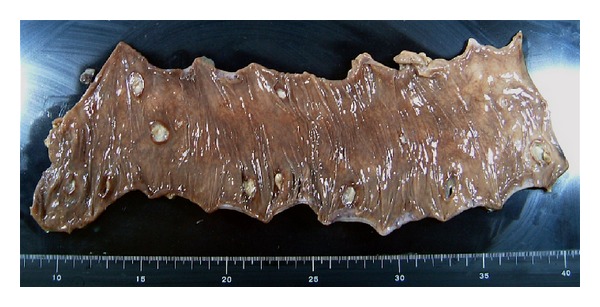
Macroscopic findings of resected transverse colonic specimen show multiple perforations. Perforated holes with circular-shape are each from 5 to 10 mm in diameter.

**Figure 3:**
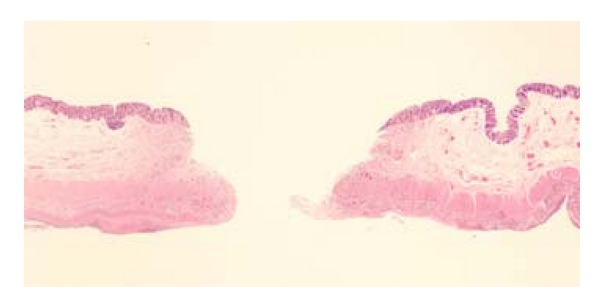
Microscopic specimen demonstrates punched-out ulcer. There is active inflammation around the ulcer. (haematoxylin and eosin ×40).

 At the time of admission to our hospital, her vital signs were as follows: body temperature 36 degrees Celsius, heart rate 81 beats/min, blood pressure 102/45 mm Hg, and respiratory rate 20/min. Physical examination revealed marked abdominal distention, with slight guarding and rebound tenderness. No bowel sounds could be heard on auscultation. She had no history of consumption of contaminated foods or water. There were no clinical features, such as rose spots, tenesmus, mucous/bloody diarrhea, or joint pain. She had sicca syndrome and dry eye, but no oral aphthae or genital ulcers. She had never visited any countries with poor sanitary conditions or tropical areas and had no history of venereal diseases. The pathergy test was negative. The results of blood investigations were as follows: white blood cells 3950/mm^3^, hemoglobin 10.3 g/dL, and platelet count 73,000/mm^3^. The serum C-reactive protein level was 3.7 mg/dL. Renal function, hepatobiliary function, serum electrolytes, and the coagulation profile were within normal limits. Radioimmunoassay for hepatitis virus markers in the serum revealed a positive test for hepatitis B surface antigen (HBs Ag) and negative tests for both hepatitis B surface antibody (HBs Ab) and anti-hepatitis C antibody. Serum tests for rheumatoid factor and HLA-B51 were negative. Both proteinase-3-antineutrophil cytoplasmic antibody (PR3-ANCA) and myeloperoxidase-antineutrophil cytoplasmic antibody (MPO-ANCA) titers were within normal limits (<10 EU). A chest X-ray obtained with the patient upright demonstrated free air under the diaphragm bilaterally (Figure 1). An emergency laparotomy was performed because of suspected panperitonitis caused by intestinal perforation. The abdominal cavity contained approximately 400 mL of fetid fluid. The stomach, duodenum, and small intestine were normal on exploration. There were several perforations on the antimesenteric aspect of the transverse colon. Therefore, a transverse colectomy and loop colostomy were performed. The resected specimen contained a large amount of soft stool and exhibited 11 punched-out ulcerations, many of which measured up to 10 mm in diameter (Figure 2). Microscopic examination revealed nonspecific findings and showed perforation of the intestinal wall with infiltration of the ulcer bed with leukocytes (Figure 3). 

 Slow-release diclofenac sodium had been discontinued since the time of admission and, subsequently, the corticosteroid was also tapered off and discontinued. On day 14, colonoscopy revealed mild aphthoid ulcers at the anastomotic site, but no evidence of perforation. The patient made good postoperative recovery. Colostomy closure was performed on day 42 without any complications, and the patient was discharged on day 73 of hospitalization.

## 3. Discussion

Multiple colonic perforations are rare, but life-threatening, and may be caused by inflammatory bowel diseases (Crohn's disease and ulcerative colitis), rheumatological disorders (Behcet's disease, SLE), medications, infectious enterocolitides, such as amebic, typhoidal, and tuberculous colitis, and neoplasms. Our patient did not fulfill the diagnostic criteria for Crohn's disease, Behcet's disease, or SLE. Vasculitis, such as microscopic polyangiitis, was thought to be unlikely because of the normal PR3-ANCA and MPO-ANCA titers and absence of renal function disorder, and in the absence of the histopathological finding of cells infiltrating the walls of small arterioles. Microscopic examination of the resected specimen revealed no pathogens. Infectious enterocolitis was considered to be unlikely because of the absence of rose spots, tenesmus, mucous/bloody diarrhea, and joint pain. Accordingly, mainly the increased topical colonic permeability by the slow-release NSAID was thought to be responsible for the development of the multiple perforations. 

 NSAIDs exert both local and systemic effects on the GI tract. Local injury to the intestinal mucosa is mainly due to the entry of the NSAIDs, especially slow-release NSAIDs, and their metabolites into the enterohepatic circulation [3]. The NSAID dosing period in our patient was relatively short, and cases of colonic perforation after even short-term diclofenac intake of under one week have been reported [4]. NSAIDs are known to damage the surface epithelial cells and increase the intestinal permeability. NSAIDs can also uncouple mitochondrial oxidative phosphorylation, which impairs the mitochondrial energy production necessary for tight junction complex integrity, leading to increased intestinal inflammation and permeability [5]. Regulation of the intestinal epithelial cell barrier is central to the development of intestinal immunity and inflammation. Tight junctions create a paracellular permeability barrier that is breached when NSAIDs cause GI injury, including increased GI permeability. Intestinal barrier function relies on the tight junctions at the apical contact areas of the intestinal epithelial cells. Tight junctions define cell polarity and regulate the paracellular flow of ions and water, which are crucial functions of acinar cells [6, 7]. In general, the transit time of the drug in the transverse colon is about 4 hours after oral intake. The dissolution rate of slow-release diclofenac sodium is 70% in 8 hours; however, this rate is also influenced by the presence of food [8]. The peak blood concentration of orally ingested slow-release diclofenac sodium is achieved from 6 to 7 hours. 

 NSAIDs cause numerous side effects, and these drugs together with corticosteroids produce additive damage to the gastrointestinal tract [9–11]. Corticosteroids have powerful immunosuppressive actions, impair the ability to contain a perforation in its early stages, and delay the consecutive healing process [12, 13]. Concomitant use of corticosteroids (odds ratio 4.4–4.7) is one of the risk factors for NSAID-related GI complications [14, 15], which have been recorded to occur 7 times more frequently in patients with perforation than in matched controls [16]. 

 Increased intestinal permeability has also been reported in autoimmune diseases such as IBD, systemic sclerosis, Behçet's syndrome, SLE, ankylosing spondylitis, and juvenile idiopathic arthritis [17–20]. Altered intestinal permeability is a well-described feature of Crohn's disease [1]. Sarcoidosis and Crohn's disease may have shared pathogenetic mechanisms [2]. There is a report of a case of sarcoidosis with gastrointestinal loss of proteins [21]. Sarcoidosis and Sjogren's syndrome, as autoimmune diseases, often coexist, since both diseases may share the same immunological profile; however, multiple intestinal perforations associated with these autoimmune diseases have not yet been reported. Sarcoidosis is a systemic disease with 90% predilection for the lungs, with gastrointestinal manifestations being rare, although any organ can be involved. The incidence of clinically recognizable gastrointestinal involvement is less than 1.0% [22]. The stomach is the most commonly involved part of the GI tract, and the colon is involved less frequently, and no case of colonic perforation has been reported. Abnormal intestinal permeability has been reported in cases of active pulmonary sarcoidosis [1]. Sjogren's syndrome is a systemic autoimmune exocrinopathy involving the salivary glands, but it can also involve almost every other part of the GI tract. Systemic vasculitic manifestations of Sjogren's syndrome have been reported in approximately 10% of patients [23]. Only one study of multiple colonic ulcers due to vasculitis associated with Sjogren's syndrome has been reported in the Japanese literature [24]. 

 It is considered that slow-release NSAIDs and corticosteroids may cause aggravation of the intestinal epithelial abnormalities in patients with sarcoidosis and Sjogren's syndrome. Close caution must be exercised when both NSAIDs and corticosteroids are prescribed to patients with autoimmune diseases, as these patients are at a higher risk of development of adverse bowel events, such as colonic perforation, than patients without autoimmune diseases.
